# Pediatric Leptospirosis: A Case Report and Review of Literature

**DOI:** 10.5152/eurasianjmed.2023.23380

**Published:** 2023-12-01

**Authors:** Hakan Doneray, Halil Keskin, Hulya Akat, Ibrahim Halil Basaslan, Perihan Serifoglu Bagatir

**Affiliations:** 1 Department of Paediatrics, Atatürk University Faculty of Medicine, Erzurum, Turkey; 2 Department of Pediatric Intensive Care, Atatürk University Faculty of Medicine, Erzurum, Türkiye; 3 Veterinary Control and Research Institute, Erzurum, Turkey

**Keywords:** Children, leptospirosis, staphylococcal toxic shock syndrome

## Abstract

Leptospirosis, a zoonosis, is characterized by a wide range of clinical and laboratory findings, varying from self-limiting infection to potentially fatal disease. Leptospirosis-related clinical manifestations, except for Weil’s disease, may often be overlooked due to their nonspecificity in children. Additionally, many health-care professionals lack awareness of leptospirosis. This paper presents a case of leptospirosis that was initially misdiagnosed as staphylococcal toxic shock syndrome. The literature on this topic is also reviewed.

## Introduction

Leptospirosis is a worldwide zoonosis caused by pathogenic spirochetes of the genus *Leptospira*. It is characterized by a wide range of clinical and laboratory manifestations varying from self-limiting infection to potentially fatal disease. The classical form of Weil’s disease is characterized by jaundice, renal involvement, and hemorrhage. However, some clinical presentations affecting the central nervous and gastrointestinal system, muscle, heart, eye, and skin may often be overlooked because they are nonspecific in children, and most health professionals lack awareness of leptospirosis. Accordingly, the number of cases in studies examining pediatric patients with leptospirosis is very limited.^1^ This paper presents a case report on leptospirosis, which was initially misdiagnosed as staphylococcal toxic shock syndrome (STSS). Additionally, it reviews the clinical and laboratory manifestations, diagnostic methods, and treatment outcomes in pediatric patients with leptospirosis.

## Case Presentation

**Figure 1.**
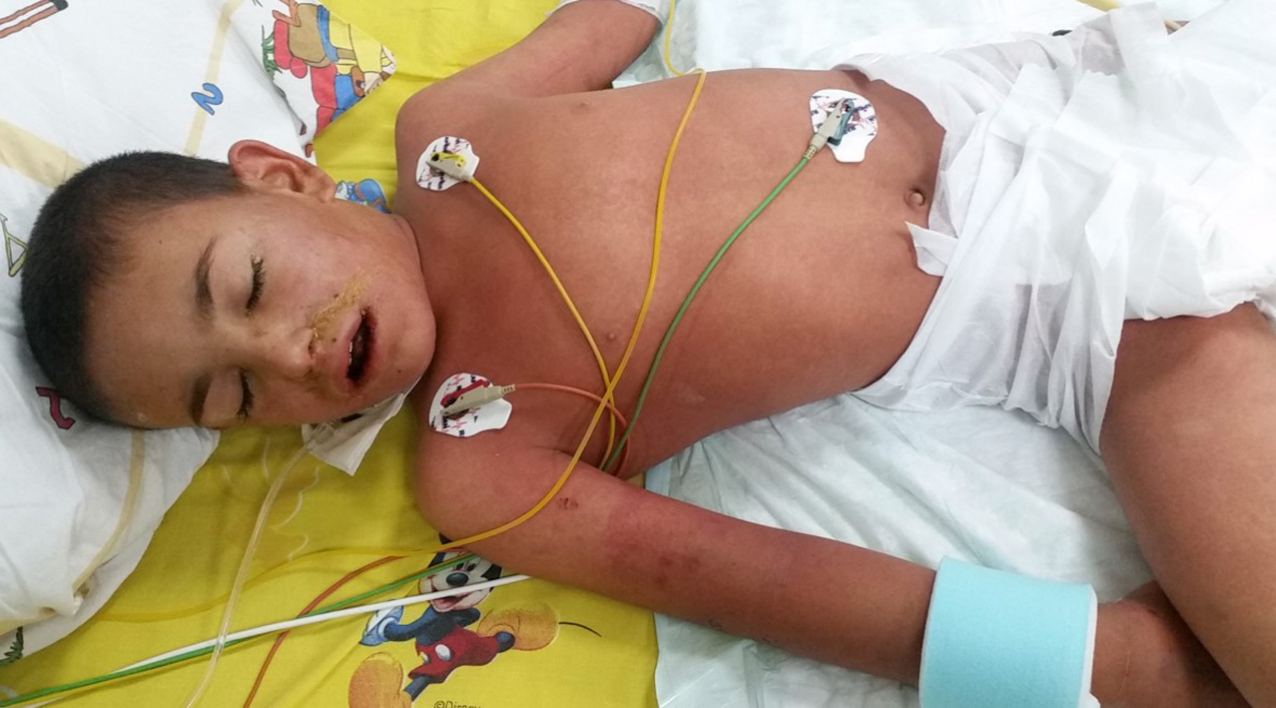
Generalized erythema of the skin.

**Figure 2.**
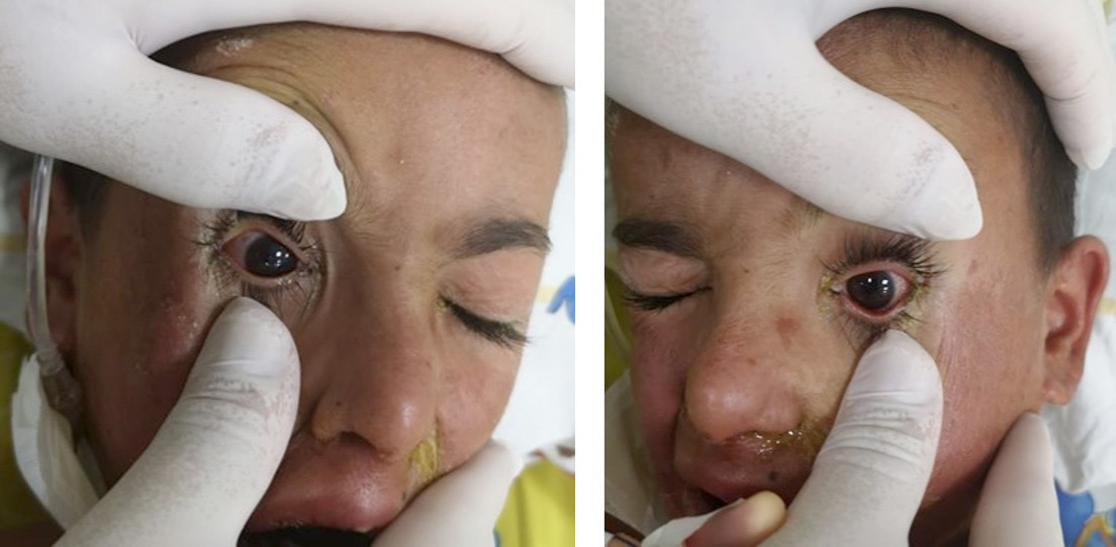
Bilateral conjunctival suffusion.

A 7-year-old boy was referred to our Paediatric Intensive Care Unit with complaints of myalgia and a generalized rash. He had experienced sudden fever, headache, and vomiting for 2 days prior to admission. The initial diagnosis was STSS, and he was being treated with vancomycin. He resided in a rural area. His prenatal, natal, postnatal, and family histories were unremarkable. On admission, the patient’s body weight and height were measured as 19 kg (10th-25th percentile) and 117.5 cm (25th percentile), respectively. He presented with a fever of 38.5°C/101.4 F, low blood pressure (82/60 mm Hg), a rapid heart rate of 169 beats/min, and rapid breathing at 27 breaths/min. The Glasgow Coma Scale score was 11 points, and capillary refill time was 3 seconds. Diffuse erythema, intraoral bleeding, bilateral conjunctival suffusion, tenderness in the calf muscles, hepatomegaly (3 cm below the right costal margin), and splenomegaly (2 cm below the left costal margin) were detected (Figures 1 and 2).

**Figure 3.**
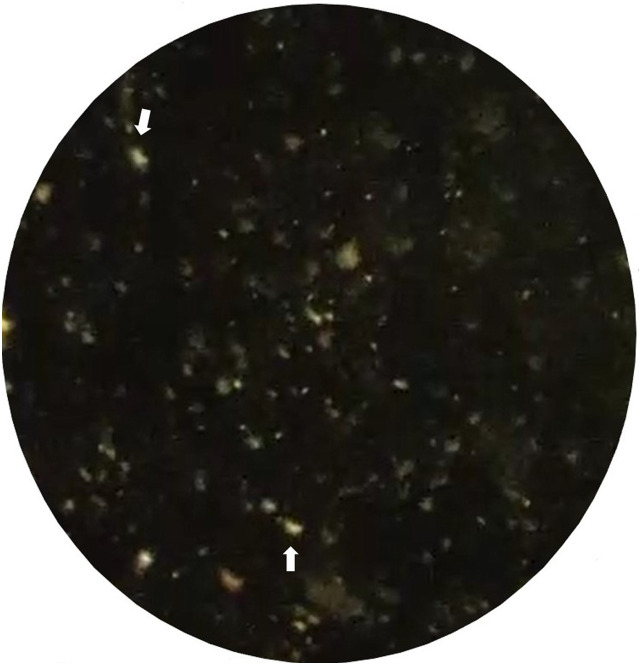
*Leptospira* on dark-ground microscopy (white arrows).

**Figure 4.**
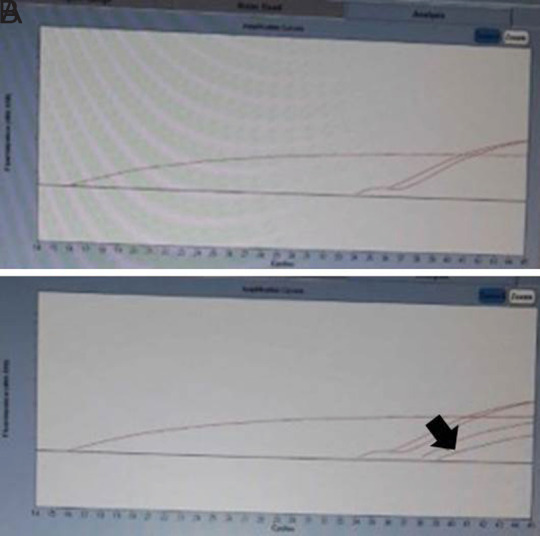
Real-time polymerase chain reaction in the urine sample. A. Control. B. Amplification curves indicating the presence of leptospiral DNA (black arrow).

The initial laboratory studies showed several abnormalities, including mild anemia, metabolic acidosis, hypoglycemia, electrolyte imbalances, hyperbilirubinemia, renal and hepatic function abnormalities, abnormal urine findings, elevated inflammatory markers, and abnormal coagulation profile (Table 1). Culture studies of blood, urine, and throat specimens did not reveal any pathogenic microorganisms. Although the microscopic agglutination test (MAT) was negative, motile spirochetes were observed in the urine sediment through dark-ground microscopy. Additionally, a real-time polymerase chain reaction (PCR) study for leptospires in a urine sample was positive (Figures 3 and 4).

**Figure 5.**
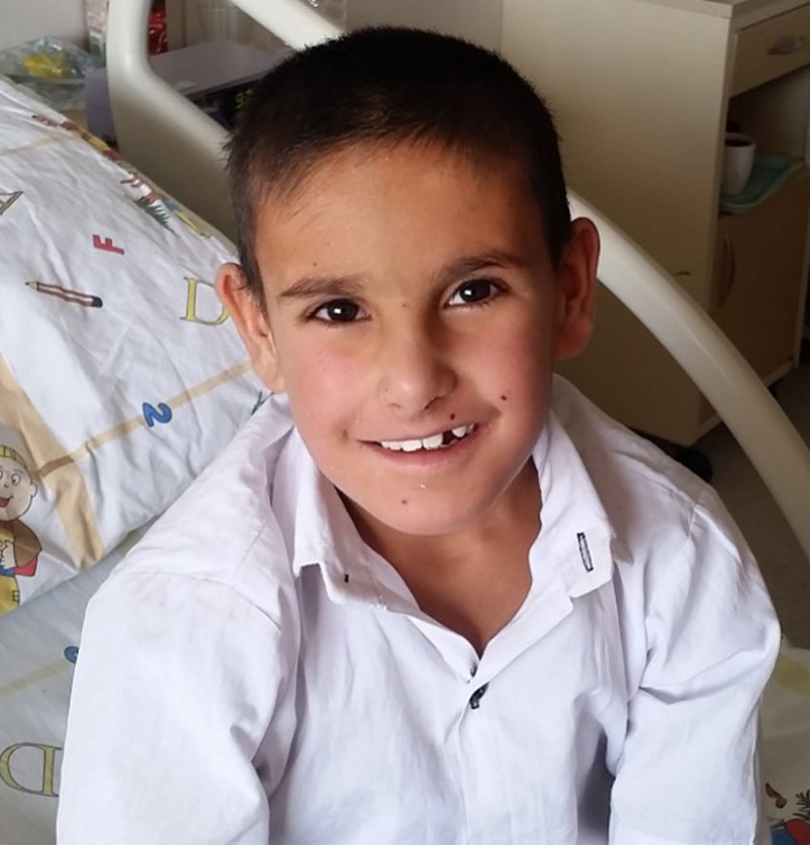
The patient’s discharge appearance.

During the first day of hospitalization, the patient received supportive therapies, including saline, adrenaline, fresh frozen plasma, albumin, and calcium gluconate. Additionally, empirical antibiotics, such as vancomycin, meropenem, and clindamycin, were administered to treat STSS and bacterial sepsis. All antibiotics were discontinued on the second day when dark-ground microscopy revealed spirochetes. Cefotaxime was started at 200 mg/kg/day in 3 doses. On the third day of hospitalization, the platelet count reached its lowest level, and most laboratory markers returned to normal. The recovery process continued consistently during the 10-day cefotaxime treatment (Table 1). The patient was discharged after making a full recovery (Figure 5). The release of this information has been authorized by the patient’s parents.

## Discussion

Leptospirosis, also known as Weil’s disease, Weil–Vasiliev disease, Stuttgart disease, swine herder’s disease, canicola fever, rice field fever, waterborne fever, nanukayami fever, cane cutter fever, swamp fever, and mud fever, is a globally important zoonotic disease with very different clinical manifestations. It is caused, so far, by 14 pathogenic species of the genus *Leptospira*. *Leptospira* are spiral-shaped, highly motile aerobic spirochetes and contain 18 or more helices per cell.^2,3^

The main reservoir for spirochetes is rodents, but they can also be carried by other mammals such as horses, dogs, cattle, sheep, goats, and pigs. *Leptospira* infection in rodents typically occurs during their infancy.^4^ They can then contaminate the environment, particularly water resources, by excreting the microorganisms in their urine throughout their lives.^5^ *Leptospira* can survive in water or muddy soil with a slightly alkaline pH for up to 6 months.^3^ *Leptospira* infection in cattle, sheep, goats, and pigs can cause signs of disease, leading to death or spontaneous abortion, although infected animals may be asymptomatic. Humans become infected when their mucous membranes or damaged skin comes into contact with contaminated water, contaminated soil, or infected animal tissues. Individuals in occupations such as farming, ranching, abattoir work, trapping, veterinary medicine, logging, sewage treatment, pet trading, and laboratory work, as well as those who participate in activities such as freshwater swimming, canoeing, kayaking, trail biking, and adventure racing, those who have pet dogs, domesticated livestock, rainwater catchment systems, and skin lesions, and those who walk barefoot through surface water and come into contact with wild rodents, are at risk of contracting leptospirosis.^6^ Leptospirosis outbreaks have been reported after floods and some triathlons where the swimming portion took place in fresh water.^7-9^ This information highlights the importance of the freshwater connection in the transmission of spirochetes to humans through contaminated water and mud. Our patient lived in a rural area, and it is possible that outdoor activities were a factor in his exposure to *Leptospira*.

Leptospirosis can occur sporadically throughout the year, but it frequently breaks out during the rainy season.^3^ It is reported that the incidence of disease in tropical regions is approximately 10 times higher than in temperate regions.^10^ However, the fact that leptospirosis is an under-reported disease- makes it difficult to obtain reliable global incidence figures. The Leptospirosis Burden Epidemiology Group of the World Health Organization estimates that there are 873 000 cases of leptospirosis worldwide annually, resulting in 48 600 deaths.^11^ Clinical reports on pediatric leptospirosis in Turkey have been rarely published. The largest clinical report to date consists of only 5 cases.^12^ To our knowledge, this is the first pediatric case diagnosed with leptospirosis in Eastern Anatolia. Although farming and cattle breeding are common in the region, the fact that this disease has not been reported in children before is an important finding that should be taken into consideration. All these findings suggest that leptospirosis may be underdiagnosed or misdiagnosed in Turkey. Possible reasons for the underdiagnosis of leptospirosis include the subclinical and self-limiting nature of most cases, lack of awareness among clinicians, and inadequate diagnostic tools in many hospitals. The clinical course of leptospirosis in children and adolescents is highly variable. The disease is usually mild and self-limiting or subclinical, but can sometimes be severe and potentially fatal.^13^ During the incubation period of 2-26 days (average 10 days), *Leptospira* multiplies in the blood and tissues. They then bind to the capillary endothelium, causing vasculitis, which is the primary cause of multisystem involvement.^14^ In pediatric patients, initial symptoms may include an abrupt onset of fever, myalgia, and headache. These symptoms have been reported in a range of 24-100% of cases (Table 2). Conjunctival suffusion, which is characterized by redness of the conjunctiva (Figure 2), is an important but oftenoverlooked sign. It was found in as many as 52% of cases in a pediatric case series.^15^ As this finding is not commonly seen in other infectious diseases, its presence in a patient with a nonspecific febrile illness should raise the possibility of leptospirosis.^16^ Gastrointestinal symptoms, including nausea, vomiting, and diarrhea, occur in 6.2-55% of cases (Table 2). Findings such as jaundice, jaundice accompanied by renal failure (Weil’s disease), liver and renal dysfunction, oliguria, hepatomegaly, lymphadenopathy, and meningitis have been reported in 2.9-64% of patients. Less common findings may include hypotension, splenomegaly, skin rash, myocarditis, and shock. Mortality rates in hospitalized pediatric patients range from 1.6% to 8% (Table 2). Although studies from disease-endemic areas have commonly reported that leptospirosis has a milder clinical course in children than in adults,^13^ our patient experienced critically severe leptospirosis, with acute kidney injury, acute hepatic dysfunction, and hemodynamic instability. Septic shock is defined as persistent hypotension requiring vasopressors to maintain mean arterial pressure ≥65 mm Hg and serum lactate >2 mmol/L (18 mg/dL) despite adequate volume resuscitation, and is very rare in pediatric series.^17^ The incidence has only been shown to be 9% in one study.^18^ However, that study did not report the findings related to shock such as hypotension, oliguria, and liver and renal dysfunctions.

There is currently no literature that specifically describes laboratory findings in children with leptospirosis. Both adult and child reports indicate that routine laboratory tests may lack specificity. The white blood cell (WBC) count can range from 3000 to 26 000/mm^3^. Thrombocytopenia or pancytopenia may appear as the initial presentation.^19,20^ An outer membrane protein of *Leptospira* is thought to interfere with the activity of the Na^+^–K^+^–Cl^−^ cotransporter in the thick ascending limb of Henle, resulting in hyponatremia and hypopotassemia that are common in severe leptospirosis.^21,22^ Proteinuria, pyuria, and granular casts are common findings on urinalysis, with occasional microscopic hematuria.^23^ Severe leptospirosis can result in renal failure. Elevated creatine kinase levels, which can be a useful indicator of the disease, have been reported in about 50% of patients with leptospirosis.^24^ Elevations in hepatic transaminases, typically not exceeding 200 IU/mL, may be observed in nearly 40% of patients. In severe cases of leptospirosis, jaundice may be observed, and in some cases, the serum bilirubin concentration may reach as high as 80 mg/dL. The analysis of cerebrospinal fluid may reveal a minimal or moderate increase in protein concentration and a normal or rarely low-glucose concentration, accompanied by a pleocytosis of lymphocytes or neutrophils.^25^ Oliguria, a WBC count above 12 900/mm^3^, repolarization abnormalities on an electrocardiogram, and alveolar infiltrates on chest radiography have been associated with an adverse outcome. The patient exhibited nearly all of the laboratory findings mentioned, except for meningeal irritation. Therefore, lumbar puncture was not performed.

Leptospirosis can be diagnosed using a range of tools, including MAT, serological methods, molecular techniques, antigen detection, and microbiologic culture. As the isolation of the organism in culture is only successful in a small percentage of cases (5-50%) and can take several weeks,^10^ other tests are necessary for early and rapid diagnosis. In suspected cases of leptospirosis, other assays are typically performed first due to the limited accessibility of the MAT. However, the use of serologic tests for acute diagnosis is limited due to the high seropositivity ratio among individuals living in endemic areas.^26^ So, serum IgM antibody titer should be measured in both acute and convalescent serum samples. A single titer of over 1 : 800 or a fourfold or greater increase in titers is considered reasonable evidence of current or recent infection with *Leptospira*. However, it should be kept in mind that serologic assays may cross-react in syphilis, relapsing fever, Lyme disease, and legionellosis.^27^ When a seropositive result is obtained, the most specific test that can be performed to detect the infecting serotype is MAT.^28,29^ During acute illness, seronegativity has been associated with cross-reactive antibodies. To diagnose leptospirosis in such cases, molecular techniques such as real-time PCR and loop-mediated isothermal amplification can be used. This is because Leptospira DNA can be detected in blood during the initial bacteremic phase of the illness, and in cerebrospinal fluid and urine a few days after the onset of symptoms. These molecular tests do not require samples from the convalescent period and do not cause any delay in the diagnosis.^30,31^ As there are no other serological tests available in our country, we conducted the MAT test that produced a negative result. This outcome may be due to either a suboptimal level of antibody titer during the acute phase or an infection with a serovar that is not included in the *Leptospira* test panel.^28,29^ We detected motile spirochetes in a urine sediment using dark-ground microscopy and also confirmed them with a real-time PCR study in urine. We think that dark-ground microscopy is a fast, easy, simple, and safe method and can be used with the real-time PCR for rapid diagnosis.

Leptospirosis may be difficult to distinguish from other infectious diseases. Acute viral illnesses, Hantavirus infection, malaria, dengue, chikungunya, scrub typhus, rickettsial diseases, typhoid fever, and ehrlichiosis should be considered in the differential diagnosis. Interestingly, our patient was referred to our intensive care unit with a preliminary diagnosis of STSS. The patient’s clinical findings may actually indicate this syndrome, which is characterized by sudden onset of fever, widespread rash, hypotension, and involvement of multiple organ systems. However, the lack of a staphylococcal infection focus in the patient contradicts the diagnosis of STSS. Half of the cases of STSS are associated with menstruation and tampon use, while the other half are related to specific factors such as surgical and postpartum wound infections, mastitis, septorhinoplasty, sinusitis, osteomyelitis, arthritis, burns, and cutaneous and subcutaneous lesions, especially in the extremities, perianal area, and axillae. Additionally, respiratory infections following influenza and enterocolitis have also been linked to STSS.^32-38^ Furthermore, conjunctival suffusion is a significant but often overlooked indicator of leptospirosis, and is not commonly seen in STSS or other infectious diseases. The presence of this finding in a nonspecific febrile illness should raise the possibility of leptospirosis.^16^ All this information emphasizes the importance of the medical history and physical examination to distinguish leptospirosis from STSS.

Most cases of leptospirosis have a self-limited course without antimicrobial therapy. However, some patients may experience severe complications that can lead to morbidity and mortality. For children with mild disease, doxycycline (2 mg/kg/day, divided into 2 doses, maximum 200 mg/day, orally for 7 days) or azithromycin (10 mg/kg, once daily, maximum 500 mg/day, on day 1 and followed by 5 mg/kg/day, once daily, maximum 250 mg/day) can be given. Hospitalized children can be treated with penicillin (250 000-400 000 units/kg/day, divided into 4-6 doses, maximum 6-12 million units/day, intravenously), doxycycline (4 mg/kg/day, divided into 2 doses, maximum 200 mg/day, intravenously), ceftriaxone (80-100 mg/kg/day, once daily, maximum 2 g/day), or cefotaxime (100-150 mg/kg/day, divided into 3-4 doses). Azithromycin can be used as an alternative drug for children who experience adverse reactions to other agents. The recommended dosage is 10 mg/kg/day, once daily, with a maximum of 500 mg/day intravenously on day 1, followed by 5 mg/kg/day, once daily, with a maximum of 250 mg/day intravenously on subsequent days. The recommended duration of treatment for severe disease is typically 7 days.^39^ Doxycycline is contraindicated for children under 8 years of age. Severe illness may require supportive care such as fluid–electrolyte therapy, blood products, ventilatory support, and renal replacement therapy.^40^ In adult patients, corticosteroids may be administered when there is pulmonary involvement and vasculitis.^41,42^ The patient received supportive care and was treated with cefotaxime, resulting in an improvement of both clinical and laboratory findings on the sixth day of admission.

The most important measures for protecting against human leptospirosis are rodent control, flood control, and avoiding potential sources of infection such as stagnant water and animal farm water runoff. Public health precautions associated with these conditions should be taken as needed.

## Conclusion

Leptospirosis is a disease that is prevalent worldwide and can be fatal. It should be considered as a possible diagnosis for patients who experience a sudden onset of fever, myalgia, and headache. Conjunctival suffusion can be useful in distinguishing it from STSS. The diagnosis of leptospirosis requires serological and molecular tests. Dark-background microscopy may serve as an alternative diagnostic tool. *Leptospira* is sensitive to penicillins, but it can still cause life-threatening complications. Therefore, it is essential to develop disease prevention strategies for both humans and animals. Increased awareness of leptospirosis among health-care professionals and easy laboratory access are necessary for these reasons.

## Figures and Tables

**Table 1.** Laboratory Characteristics During the Follow-up Period.

**Markers**	**On Admission**	**On the Third Day**	**On the Sixth Day**
Hematologic			
White blood cell (mm^3^) (4500-13 500)	12 540	10 880	15 630
Neutrophil (mm^3^) (1800-8000)	11 490	6330	9240
Lymphocyte (mm^3^) (1662-3448)	210	3530	4500
Monocyte (mm^3^) (350-400)	540	810	1300
Eosinophil (mm^3^) (<230)	270	130	580
Basophil (mm^3^) (<40)	30	80	10
Hb (g/dL) (>12)	11.7	9.4	8.7
Platelet (mm^3^) (150 000-450 000)	192 000	52 000	251 000
Venous blood gas			
pH (7.35-7.45)	7.33	7.39	7.42
PCO_2 _(mm Hg) (35-45)	19.1	44.1	42
HCO_3_ (mmol/L) (17-23)	10	26.7	28.3
Lactate (mmol/L) (1.1-2.3)	2.4	1.1	1.7
Biochemical			
Glucose (mg/dL) (70-100)	38	103	95
BUN (mg/dL) (5-18)	41.5	7.9	7
Creatinine (≤0.8)	0.9	0.2	0.15
Uric acid (mg/dL) (2.5-5.5)	9.7	2.3	1.4
Na (mmol/L) (135-145)	128	140	137
Cl (mmol/L) (95-108)	100	99	96
K (mmol/L) (3.5-5.5)	3.3	2.5	4.1
Ca (mg/dL) (8.8-10.8)	7.2	8	8.5
P (mg/dL) (4.5-5.5)	4	2.9	3.6
Mg (mg/dL) (1.7-2.2)	2.1	1.5	1.6
Total bilirubin (mg/dL) (<1.5)	3.0	2.3	0.9
Direct bilirubin (mg/dL) (0.0-0.3)	2.0	1.1	0.3
Total protein (g/dL) (4.3-7.6)	3.8	5	5.8
Albumin (g/dL) (3-5)	1.9	2.8	3.0
ALT (U/L) (3-30)	145	73	32
AST (U/L) (15-40)	88	66	26
LDH (U/L) (60-170)	425	369	387
GGT (U/L) (0-23)	61	49	29
ALP (U/L) (107-213)	143	144	128
CK (U/L) (0-70)	519	263	84
CK-MB (U/L) (1-24)	–	30	24
Urine			
Density (1012-1020)	1012	–	1015
Blood (negative)	Trace	–	Negative
Protein (negative)	Negative	–	Negative
Leucocyte esterase (negative)	Trace	–	Negative
Ketone (negative)	+1	–	Negative
Bilirubin (negative)	+1	–	Negative
Inflammatory			
CRP (mg/dL) (0-5)	260.4	220	35.8
ESR (mm/h) (0-20)	40	–	–
Procalcitonin (ng/mL) (<0.5)	84.3	–	–
Coagulation			
PT (s) (11-16.5)	30.2	16	16.7
aPTT (s) (26-34)	33.9	29.5	30.5
INR (0.8-1.2)	2.4	1.23	1.28
Fibrinogen (mg/dL) (200-400)	436	341	350
D-Dimer (ng/mL) (0-500)	7699	6330	3596

ALT, alanine transaminase; ALP, alkaline phosphatase; aPTT, activated partial thromboplastin time; AST, aspartate transaminase; BUN, blood urea nitrogen; Ca, calcium; Cl, chloride; CK, creatine kinase; CK-MB, creatine kinase, myocardial band; CRP, C-reactive protein; ESR, erythrocyte sedimentation rate; GGT, gamma glutamyl transferase; Hb, hemoglobin; HCO^3^, bicarbonate; INR, international normalized ratio; K, potassium; LDH, lactate dehydrogenase; Mg, magnesium; Na, sodium; P, phosphorus; PCO_2_, partial carbon dioxide pressure; PT, prothrombin time.

Normal values for the patient’s age are provided in parentheses.

**Table 2.** Our Case and the Studies Including a Large Number of Pediatric Patients with Leptospirosis.

Clinical Characteristics	Our Patient, Turkey	Tomari K,^15^ 2018 Japan (n = 44) n (%)	Narayanan R,^43^ 2016 India (n = 35) n (%)	Pérez-García J,^44^ 2016, Colombia (n = 74) n(%)	Guerrier G,^45^ 2013, New Caledonia (n = 60) n(%)	Spichler A,^13^ 2012, Brazil (n = 42) n (%)	Agesilas F,^46^ 2005, Reunion Island (n = 16) n (%)	Rajajee S,^18^ 2002, India (n = 139) n (%)	Cruz ML,^47^ 1994, Brazil (n = 23) n (%)
Age	7 Years	0-20 Years	0-17 Years	5-17 Years	6-17 Years	< 18 Years	9-17 Years	5-17 Years	4-12 Years
Fever	Yes	42 (96)	35 (100)	Unknown	33 (55)	Unknown	15 (93.7)	133 (96)	23 (100)
Headache	Yes	Unknown	29 (82.9)	Unknown	43 (71.6)	Unknown	Unknown	34 (24)	12 (52.1)
Myalgia	Yes	23 (52)	22 (62.9)	18 (24.3)	43 (71.6)	Unknown	Unknown		16 (69.5)
Gastrointestinal symptoms	No	15 (34)	12 (34.2)	31 (41.8)	33 (55)	Unknown	1 (6,2)	Unknown	8 (34.8)
Respiratory symptoms	No	Unknown	17 (48.6)	4 (5.4)	18 (30)	13/39 (33)	1 (6.2)	Unknown	Unknown
Conjunctival suffusion	Yes	23 (52)	9 (25.7)	Unknown	13 (21.6)	Unknown	1 (6.2)	21 (15)	3 (13)
Hypotension	Yes	0 (0)	0 (0)	0 (0)	0 (0)	Unknown	2 (12.5)	Unknown	Unknown
Rash	Yes	1 (2)	Unknown	8 (10,8)	Unknown	Unknown	Unknown	Unknown	Unknown
Jaundice	No	6 (14)	11 (31.4)	15 (20.2)	11 (18.3)	26/41 (64)	7 (43)	25 (18)	11 (47.8)
Lymphadenopathy	No	2 (5)	Unknown	15 (20.2)	Unknown	Unknown	Unknown	Unknown	Unknown
Hepatomegaly	Yes	Unknown	19 (54.3)	12 (16.2)	Unknown	Unknown	Unknown	100 (72)	1 (4,3)
Splenomegaly	Yes	Unknown	Unknown	1 (1.3)	Unknown	Unknown	Unknown	Unknown	Unknown
Oliguria	Yes	Unknown	Unknown	Unknown	9 (15)	16/41 (39)	Unknown	Unknown	Unknown
Meningitis	No	4 (9)	1 (2.9)	Unknown	Unknown	Unknown	4 (25)	10 (7)	Unknown
Myocarditis	No	Unknown	3 (8.6)	Unknown	1 (1.6)	Unknown	1 (6.2)	9 (7)	Unknown
Liver dysfunction	Yes	12 (27)	Unknown	Unknown	Unknown	Unknown	9 (56)	Unknown	Unknown
Renal dysfunction	Yes	11 (25)	Unknown	Unknown	Unknown	Unknown	8 (50)	Unknown	4 (17.4)
Shock	Yes	0 (0)	0 (0)	Unknown	0 (0)	Unknown	0 (0)	12 (9)	Unknown
Weil’s disease (jaundice and renal involvement)	Yes	Unknown	Unknown	Unknown	7 (11.6)	Unknown	6 (37.5)	Unknown	Unknown
Death	No	0 (0)	0 (0)	0 (0)	1 (1.6)	2 (5)	0 (0)	8 (6)	0 (0)
